# Disease foci of pharmaceutical research and development as reflected in applications for International Nonproprietary Names, 1953–2022

**DOI:** 10.2471/BLT.23.291203

**Published:** 2024-11-04

**Authors:** Sarel F Malan, Sophie AJ Lasseur, Antonio Romeo, Raffaella Balocco

**Affiliations:** a INN Programme and Classification of Medical Products, World Health Organization, 20 Avenue Appia, 1211 Geneva, Switzerland.

## Abstract

**Objective:**

To evaluate trends in pharmaceutical research and development, and to correlate these trends with global medical need.

**Methods:**

We obtained details of proposed pharmaceutical substances from 1953 to 2022 from the International Nonproprietary Names (INN) database. We used the DrugBank and Cortellis databases to obtain the INN included in approved medicines over the same period. To evaluate trends, we categorized INN into 12 therapeutic classes according to their stem classification, and compared these trends with actual global medical need by extracting the INN in medicines included in essential medicines lists.

**Findings:**

Out of a total of 10 611 proposed INN within our 12 therapeutic groups, 2280 were included in approved or registered medicines. We observed a considerable decrease in the number of new INN for anti-infective and antiparasitic, central nervous system and cardiovascular system medicines over the study period. In contrast, the number of new substances in the fields of antineoplastic, immunomodulatory, blood and haemopoietic system, and cell and gene therapy medicines has been increasing. In terms of public health impact, only 17.3% (441/11 453) of all INN in approved medicines are included in the *World Health Organization Model list of essential medicines*, the highest proportion of which are anti-infective and antiparasitic medicines.

**Conclusion:**

Despite a high demand from global health systems, medicine development for neglected tropical and other infectious diseases remains largely dependent on national policy, governmental and philanthropic funding, and partnerships. Better alignment of research and development strategy and investment in global medical needs is required.

## Résumé

**Objectif:**

Identifier les tendances en matière de recherche et développement pharmaceutique, et établir des liens entre ces tendances et les besoins médicaux mondiaux.

**Méthodes:**

Nous avons prélevé, dans la base de données des Dénominations Communes Internationales (DCI), les détails relatifs aux substances pharmaceutiques proposées entre 1953 et 2022. Nous avons exploré les bases de données DrugBank et Cortellis afin d'obtenir les DCI mentionnées dans les médicaments autorisés durant la même période. Enfin, pour identifier les tendances, nous avons réparti les DCI en 12 catégories thérapeutiques en fonction de leur classification d'origine, puis comparé ces tendances avec les besoins médicaux réels dans le monde, en extrayant les DCI des substances qui figurent dans la liste des médicaments essentiels.

**Résultats:**

Sur un total de 10 611 DCI proposées au sein de nos 12 catégories thérapeutiques, 2280 étaient liées à des médicaments autorisés ou enregistrés. Nous avons observé une baisse considérable du nombre de nouvelles DCI pour les médicaments anti-infectieux et antiparasitaires, ainsi que pour les médicaments pour le système nerveux central et le système cardiovasculaire au cours de la période étudiée. En revanche, le nombre de nouvelles substances dans des domaines tels que les médicaments antinéoplasiques et immunomodulateurs a augmenté, mais aussi les médicaments pour le système sanguin et hématopoïétique ainsi que les thérapies cellulaires et géniques. En termes d'impact sur la santé publique, à peine 17,3% (441/11 453) des DCI intégrées dans les médicaments autorisés sont reprises dans *la Liste modèle des médicaments essentiels de l'Organisation mondiale de la Santé,* la plupart d'entre elles étant des anti-infectieux et des antiparasitaires.

**Conclusion:**

Malgré la forte demande de la part des systèmes de santé mondiaux, le développement de médicaments destinés à soigner des maladies tropicales négligées et d'autres maladies infectieuses dépend encore largement de la politique nationale, du financement des gouvernements et organismes philanthropiques, ainsi que des partenariats. Il est donc nécessaire d'harmoniser la stratégie de recherche et développement et les investissements relatifs aux besoins médicaux dans le monde.

## Resumen

**Objetivo:**

Evaluar las tendencias de la investigación y el desarrollo farmacéuticos y correlacionarlas con las necesidades médicas a nivel mundial.

**Métodos:**

Se obtuvieron detalles de las sustancias farmacéuticas propuestas desde 1953 hasta 2022 de la base de datos de Denominaciones Comunes Internacionales (DCI). Se utilizaron las bases de datos DrugBank y Cortellis para obtener las DCI incluidas en medicamentos aprobados durante el mismo periodo. Para evaluar las tendencias, se clasificaron las DCI en 12 clases terapéuticas según la clasificación de su origen, y se compararon estas tendencias con las necesidades médicas mundiales reales a partir de las DCI de los medicamentos incluidos en las listas de medicamentos esenciales.

**Resultados:**

De un total de 10 611 DCI propuestas dentro de los 12 grupos terapéuticos, 2280 se incluyeron en medicamentos aprobados o registrados. Se observó una disminución considerable del número de DCI nuevas para medicamentos antiinfecciosos y antiparasitarios, del sistema nervioso central y del sistema cardiovascular durante el periodo de estudio. Por el contrario, el número de sustancias nuevas en los ámbitos de los medicamentos antineoplásicos, inmunomoduladores, para el sistema sanguíneo y hematopoyético y para la terapia celular y génica ha ido en aumento. En términos de impacto en la salud pública, solo el 17,3% (441/11 453) de todas las DCI en medicamentos aprobados están incluidas en *la lista modelo de medicamentos esenciales de la Organización Mundial de la Salud,* siendo el mayor porcentaje de ellas medicamentos antiinfecciosos y antiparasitarios.

**Conclusión:**

A pesar de la gran demanda de los sistemas sanitarios mundiales, el desarrollo de medicamentos para las enfermedades tropicales desatendidas y otras enfermedades infecciosas sigue dependiendo en gran medida de la política nacional, la financiación gubernamental y filantrópica y las asociaciones. Es necesario alinear mejor la estrategia de investigación y desarrollo y la inversión en las necesidades médicas a nivel mundial.

## ملخص

**الغرض:**

تقييم اتجاهات البحث والتطوير الدوائي، وربط هذه الاتجاهات بالاحتياجات الطبية العالمية.

**الطريقة:**

حصلنا على تفاصيل المواد الدوائية المقترحة من عام 1953 إلى عام 2022 من قاعدة بيانات الأسماء الدولية غير مسجلة الملكية (INN). واستخدمنا قاعدتي البيانات DrugBank وCortellis للحصول على الأسماء الدولية غير مسجلة الملكية المضمنة في الأدوية المعتمدة خلال نفس الفترة. لتقييم الاتجاهات، قمنا بتصنيف الأسماء الدولية غير مسجلة الملكية إلى 12 فئة علاجية وفقًا لتصنيفها الأساسي، وقارنا هذه الاتجاهات مع الاحتياجات الطبية العالمية الفعلية من خلال استخراج الأسماء الدولية غير مسجلة الملكية في الأدوية المدرجة في قوائم الأدوية الأساسية.

**النتائج:**

من إجمالي 10611 اسمًا دوليًا غير مسجل الملكية مقترحًا ضمن المجموعات العلاجية البالغ عددها 12 لدينا، تم تضمين 2280 اسمًا دوليًا غير مسجل الملكية في الأدوية المعتمدة أو المسجلة. لقد لاحظنا انخفاضًا ملموسًا في عدد الأسماء الدولية غير مسجلة الملكية الجديدة للأدوية المضادة للعدوى والطفيليات، وأدوية الجهاز العصبي المركزي، وأدوية القلب والأوعية الدموية خلال فترة الدراسة. وفي المقابل، فإن هناك ازدياد في عدد المواد الجديدة في مجالات الأدوية المقاومة للأورام، والتعديل المناعي، ونظام الدم ومكونات الدم، والعلاج الخلوي والجيني. ومن حيث التأثير على الصحة العامة، فإن %17.3 فقط (441/11453) من جميع الأسماء الدولية غير مسجلة الملكية في الأدوية المعتمدة، مدرجة في قائمة منظمة الصحة العالمية النموذجية للأدوية الأساسية، وأعلى نسبة منها هي أدوية مضادة للعدوى والطفيليات.

**الاستنتاج:**

بالرغم من الطلب المرتفع من جانب أنظمة الصحة العالمية، فإن تطوير الأدوية للأمراض الاستوائية المهملة، والأمراض المعدية الأخرى يظل معتمدًا بشكل كبير على السياسة الوطنية، والتمويل الحكومي والخيري، والشراكات. وهناك حاجة إلى تنسيق أفضل لاستراتيجية البحث والتطوير والاستثمار في الاحتياجات الطبية العالمية.

## 摘要

**目的:**

旨在评估药物研发的趋势，并将这些趋势与全球医疗需求关联起来。

**方法:**

我们从国际非专利名称 (INN) 数据库中获得了 1953 年至 2022 年期间拟议药物的详细信息。我们使用 DrugBank 和 Cortellis 数据库来获取同期获批药物中包含的 INN。为了评估趋势，我们根据其词干分类将 INN 分为 12 个治疗类别，并通过提取基本药物清单中所含药物的 INN，将这些趋势与全球实际医疗需求进行了对比。

**结果:**

我们的 12 个治疗组共包含 10,611 个拟议 INN，其中 2,280 个被纳入了已获批或已注册药物。据我们观察，在研究期间，针对抗感染和抗寄生虫药物以及中枢神经系统和心血管系统相关药物的新 INN 数量大幅减少。相比之下，抗肿瘤、免疫调节、血液和造血系统领域的新药以及细胞和基因治疗药物的数量却在持续增加。从公共卫生影响的角度来看，仅有 17.3% (441/11,453) 的已获批药物 INN 被列入了世界卫生组织基本药物示范清单，其中抗感染和抗寄生虫药物所占比例最高。

**结论:**

尽管全球卫生系统的需求很高，但是针对不受重视的热带和其他传染性疾病的药物开发在很大程度上仍然取决于国家政策、政府和慈善基金以及伙伴关系。需要加强统筹以确保研发战略和全球医疗需求相关投资之间的协调一致性。需要加强统筹以确保研发战略和全球医疗需求相关投资之间的协调一致性。

## Резюме

**Цель:**

Оценить тенденции в области фармацевтических исследований и разработок и соотнести эти тенденции с глобальными медицинскими потребностями.

**Методы:**

Из базы данных международных непатентованных названий (МНН) были получены сведения о предлагаемых фармацевтических субстанциях за период с 1953 по 2022 год. Для получения информации о МНН, включенных в список одобренных лекарственных препаратов за тот же период, использовались базы данных DrugBank и Cortellis. Для оценки тенденций МНН были распределены по 12 терапевтическим классам в соответствии с классификацией STEM, а затем было проведено сравнение этих тенденций с фактической глобальной медицинской потребностью путем извлечения МНН из лекарств, включенных в списки основных лекарственных средств.

**Результаты:**

Из 10 611 предложенных МНН в 12 терапевтических группах 2280 были включены в список одобренных или зарегистрированных лекарственных средств. За период исследования отмечено значительное снижение количества новых МНН для противоинфекционных и противопаразитарных препаратов, препаратов, влияющих на центральную нервную систему и сердечно-сосудистую систему. Напротив, количество новых веществ в области противоопухолевых, иммуномодулирующих препаратов, препаратов для лечения крови и гемопоэтической системы, а также клеточной и генной терапии постоянно растет. С точки зрения воздействия на общественное здравоохранение, только 17,3% (441/11 453) всех МНН утвержденных лекарственных средств включены в типовой список основных лекарственных средств ВОЗ, причем наибольшую долю среди них составляют противоинфекционные и противопаразитарные препараты.

**Вывод:**

Несмотря на высокий спрос со стороны глобальных систем здравоохранения, разработка лекарств для лечения забытых тропических и других инфекционных заболеваний по-прежнему во многом зависит от национальной политики, государственного и филантропического финансирования и партнерских отношений. Необходимо обеспечить более четкое соответствие между стратегией исследований и разработок и инвестициями в глобальные медицинские потребности.

## Introduction

Neglected diseases, especially neglected tropical diseases, are prevalent in countries and communities that can least afford health care, leading to the restricted development and availability of medicines in these fields. Between 1975 and 2004, only 21 of 1556 approved medicines were for neglected tropical diseases, malaria or tuberculosis.^1^^,^^2^ From 2000 to 2011, 37 of the 850 new products to market targeted neglected diseases, comprising 25 products with a new indication or formulation, eight vaccines and only four new chemical entities. Of a total of 1 523 259 disability-adjusted life years counted during this period, neglected diseases accounted for 10.5% (159 976) (malaria and tuberculosis accounting for 33 976 and 34 217, respectively, or 2.2% each), with other infectious and parasitic diseases accounting for 11.9% (181 441).^3^ The limited development in this field is often attributed to the unfavourable cost-to-risk ratio in drug research and development, leading to the abandonment of potentially effective medicines for economic reasons.^4^ Oral eflornithine for sleeping sickness is an example of a drug that would currently be unavailable if not for specific initiatives and campaigns in the 1990s.^5^^,^^6^

Despite progress in treating the 20 neglected tropical diseases or disease groups listed by the World Health Organization (WHO), 1.65 billion people still required treatment for these diseases in 2021.^7^ The most prevalent of these diseases are soil-transmitted helminth infections, lymphatic filariasis, schistosomiasis, scabies, leishmaniasis, Chagas disease and dengue.^8^ Protozoan and helminthic parasites cause more than 50% of neglected tropical diseases, followed in number by bacterial, viral and fungal infections;^9^ however, the treatment of many of these diseases is based on medicines approved decades ago.^10^

Antimicrobial resistance is fast becoming a global challenge that could cause 2.4 million deaths by 2050.^11^^,^^12^ Fungal infections, responsible for an estimated 1.5 million deaths annually, remain neglected.^13^^–^^15^ Amphotericin B, a polyene that was developed in the 1950s, remains the preferred treatment for cryptococcal meningitis and mycosis.^16^

In addition to the above-mentioned diseases, noncommunicable diseases have surged in low- and middle-income countries. Noncommunicable diseases account for 17 million premature global deaths in those younger than 70 years; most of these deaths occur in low- and middle-income countries.^17^ However, these diseases have received minimal attention and only a small share of international health aid.^18^

Despite these challenges, several successes are evident as a result of the global drive to address unmet medical need.^19^ These include the development of the human papillomavirus vaccine, treatments for melanoma and hepatitis C, and the rapid deployment of messenger RNA technology for coronavirus disease vaccines.^20^

**Fig. 1:**
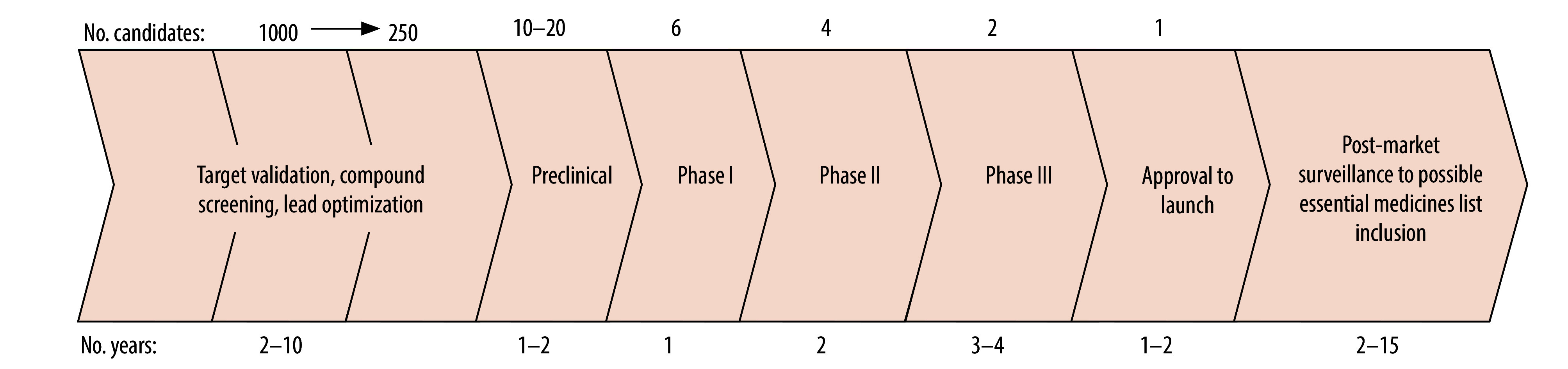
Drug development pipeline, depicting the various stages with approximate time and number of candidates involved

In this paper, we used the International Nonproprietary Name (INN) database, coordinated by WHO since 1953, as a proxy for drug research and development trends in specific therapeutic areas over the past seven decades. Because an INN is required by regulatory authorities before an application for registration of a drug can commence, INN applications for new pharmaceutical substances are typically submitted during phase I or early phase II clinical trials in the drug development pipeline (Fig. 1).^21^ Each INN is allocated following a systematic process and contains a stem (mostly a suffix) and a fantasy prefix. Stems are defined based on mechanism of action as submitted by the applicant or, in some cases, structural features, and each stem is included under a specific stem classification according to general pharmacological classes.^22^^–^^24^ In this paper, we aim to evaluate and correlate trends in pharmaceutical research and development with global medical need, as determined from essential medicines lists reflecting global and regional disease burden.

INN: International Nonproprietary Name.

Notes: the cumulative time from development to approval and possible inclusion in an essential medicines list is at least 12–15 years. New INN application typically occurs during phase I or early in phase II.

## Methods

We obtained information on all proposed INN, from inception of the programme until the end of 2022 (up to and including WHO INN proposed list 128),^25^ from the INN integrated data management system database, including: list number and year; anatomical therapeutic classification (if available); WHO stem classification (e.g. S520 equates to antimycobacterials and H400 to antihyperlipidaemic drugs); and a mechanism of action and use statement.^24^ We combined the selected WHO stem classes into 12 groups for evaluation, namely: analgesic and anti-inflammatory; central nervous system (not classified as analgesic); anti-infective and antiparasitic; antineoplastic; immunomodulatory (not specifically indicated for cancer); cardiovascular; blood and haemopoietic system; respiratory and anti-allergy; gastrointestinal and urinary tract; metabolism, and water and mineral homeostasis; hormones; and cell and gene therapy (when not included in any of the previous groups). 

We searched the DrugBank database and the Cortellis Partner Program on Clarivate Web of Science for products that have been registered, approved or launched (referred to throughout as approved) since 1953.^26^^,^^27^

We used the 22nd *WHO Model list of essential medicines* (2021)^28^ to obtain a primary list of INN in approved medicines that are included in essential medicines lists. We also obtained a combined list of approved medicines from the 2017 WHO model list and the essential medicines lists or national formularies of 137 countries (referred to throughout as the global list) from a published article,^29^ from which we derived information on global and country-specific essential medicines.

We conducted a technical check of all names obtained from external sources, and corrected INN for spelling, the addition or deletion of salt forms, and the use of national or common names instead of the appropriate INN; for example, we changed mesalamine to mesalazine and tifomycin to chloramphenicol. For the essential medicines list, we obtained the anatomical therapeutic chemical classification (of which there are 14 at the first level) from the 2023 index when included;^30^ when this classification code was not available, we deduced one according to the guidelines^30^ up to the second or third level to enable evaluation and comparisons in the essential medicines list data set.

We transferred all data to Access (Microsoft Corporation, Redmond, United States of America) and categorized them into our 12 main therapeutic groups of interest according to the INN stem classification.^24^^,^^25^ We sorted the essential medicines list and global list data according to the 14 anatomical therapeutic chemical classification main groups (where available) as well as the INN stem classification groups. In all data sets, we grouped the proposed INN within the seven decades from 1953 to 2022.

## Results

### Proposed INN and INN in approved medicines

**Fig. 2:**
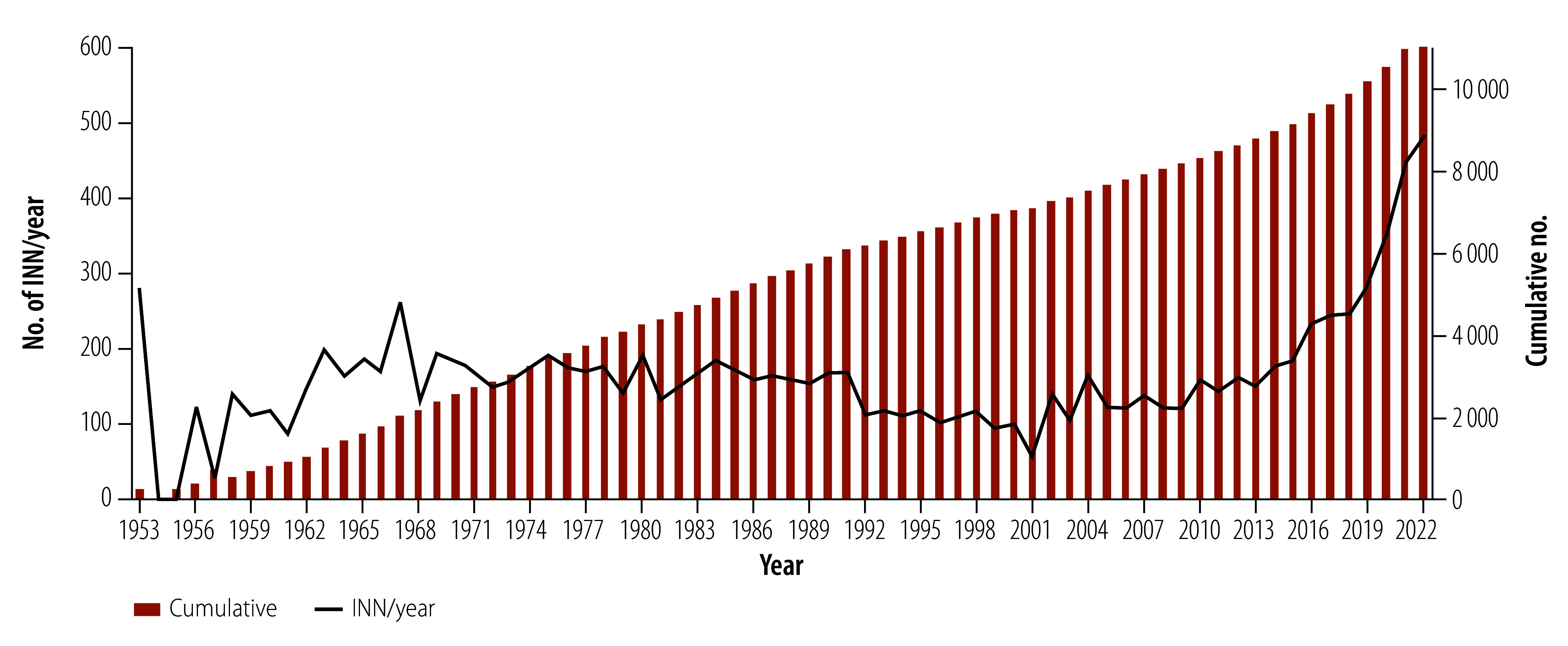
The number of INN proposed between 1953 and 2022

We obtained 11 453 unique proposed INN, with anatomical therapeutic chemical classification available for 2897.^24^ The number of pharmaceutical substances proposed per decade ranged from 968 in 1953–1962 to 2642 in 2013–2022 (Fig. 2). The total data set for the 12 selected therapeutic groups included 10 611 proposed INN, with the other 842 INN comprising miscellaneous compounds such as excipients, detergents, diagnostic aids, sunscreens, chelating agents and detoxicants. Central nervous system (16.9%; 1790), antineoplastic (16.7%; 1773), anti-infective and antiparasitic (15.0%; 1592) and cardiovascular (12.4%; 1312) compounds comprised 60.9% (6467) of the 10 611 INN included.

INN: International Nonproprietary Name.

**Fig. 3:**
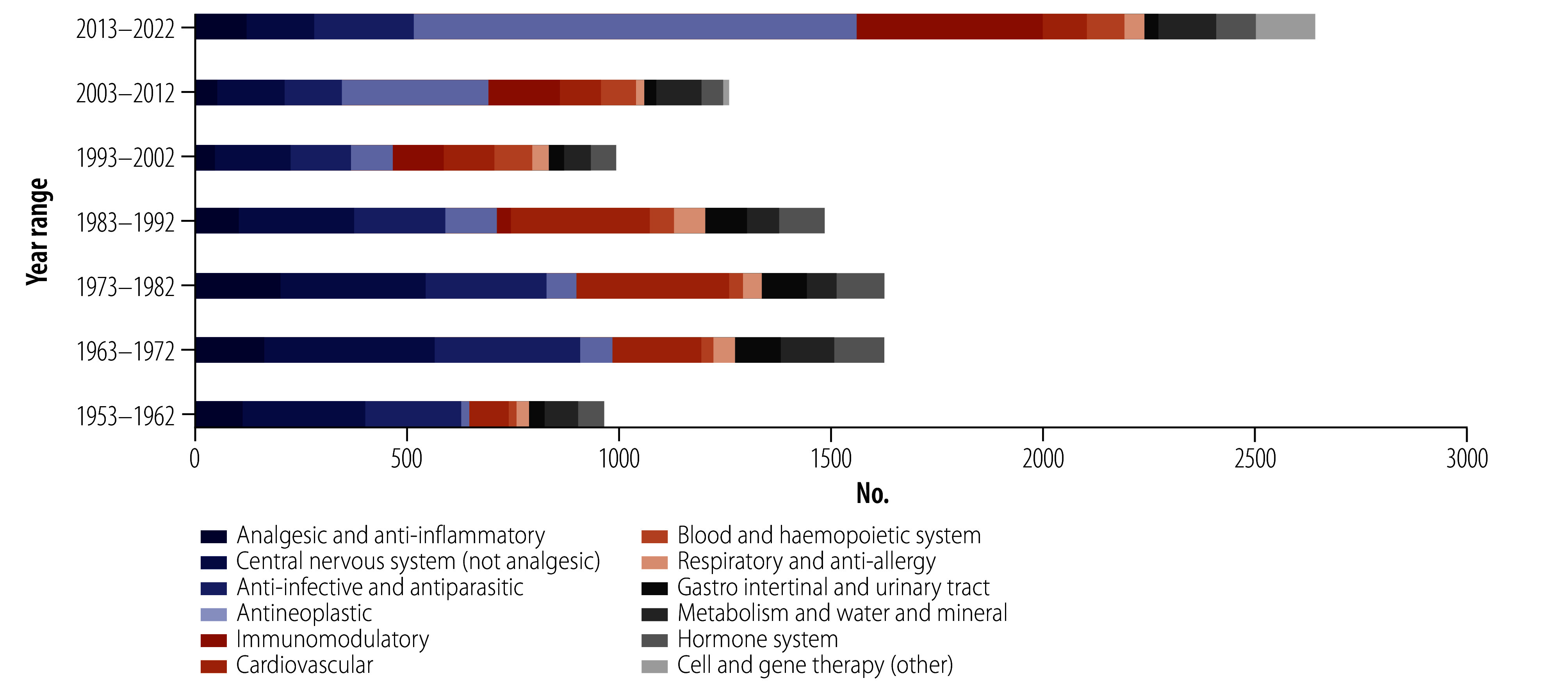
The changing composition of proposed INN as obtained from its integrated database, in 12 categories based on the INN stem classification, 1953–2022

Clear trends in drug development and discovery are evident (Fig. 3). In the first three decades, central nervous system, anti-infective and antiparasitic, cardiovascular, and analgesic and anti-inflammatory substances featured prominently, comprising 71.8% (3032/4223) of all proposed INN. Central nervous system (29.8%; 288/968) and anti-infective and antiparasitic (23.5%; 227/968) substances represented the highest proportions in the first decade of the INN Programme, while cardiovascular substances grew in prominence to a high of 22.0% (685/3116) during 1973–1992. However, this picture changed substantially in the decades that followed: of the total number of INN proposed during 2013–2022, these four groups comprised only 23.5% (620/2642) while antineoplastic and immunomodulatory substances comprised 56.3% (1485/2640). 

INN: International Nonproprietary Name.

INN proposed for substances affecting metabolism and water and mineral homeostasis remained relatively constant throughout the study period; 34.8% (230/660) of the substances in this group were indicated for carbohydrate metabolism.

In the last decade of study (2013–2022), monoclonal antibodies (mAbs) and kinase inhibitors comprised at least 62.3% (650/1044) of INN proposed in the field of antineoplastic drug development, comprising 292 naked mAbs, 83 mAb-conjugates and 275 kinase inhibitor new INN.

**Fig. 4:**
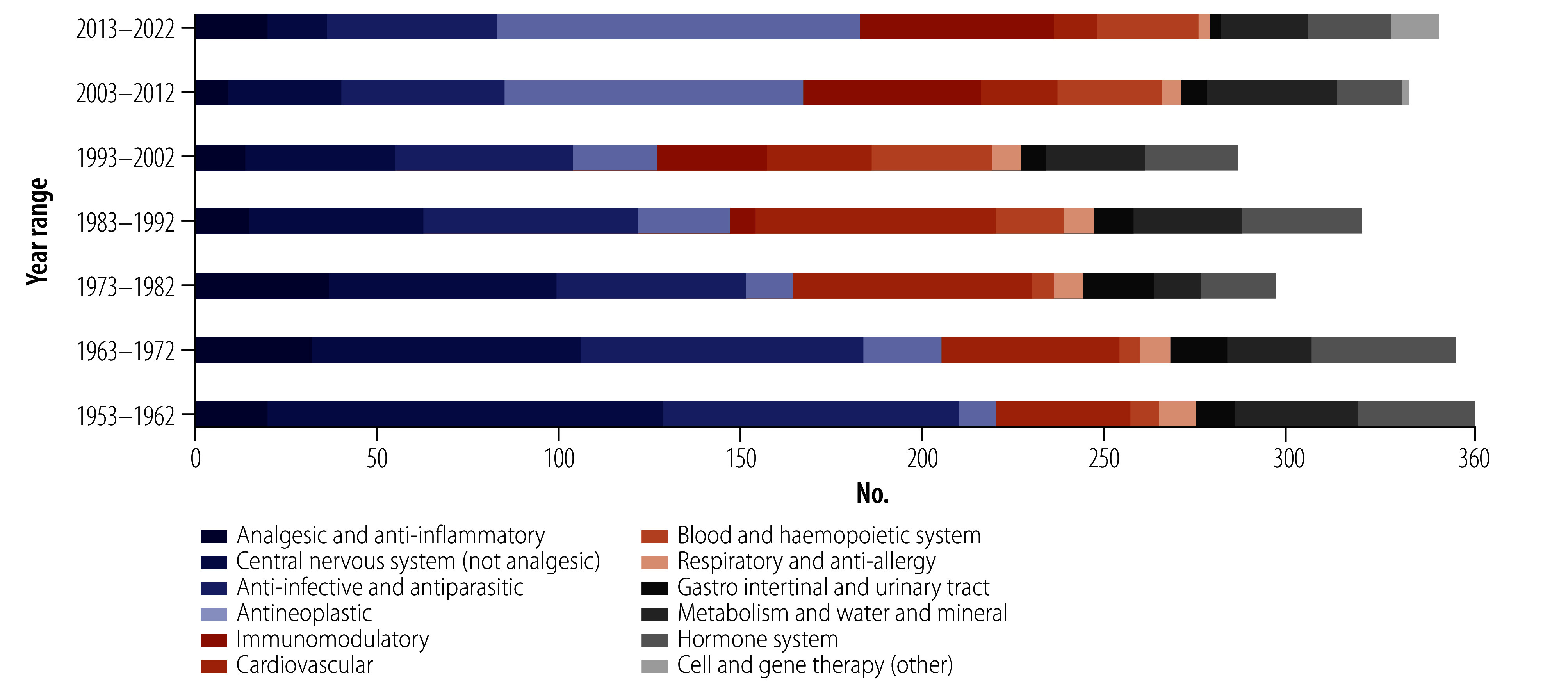
The changing composition of INN of pharmaceutical substances included in registered or approved medicinal products, in 12 categories based on the INN stem classification, 1953–2022

From the DrugBank and Cortellis databases, we found 2343 and 1235 unique INN in approved medicines, respectively.^26^^,^^27^ We combined these results and eliminated duplicates to yield 2549 unique INN, 2280 of which were included in our selected therapeutic groups over the study period (Fig. 4).^26^^,^^27^ Amongst others, we observed a reduction in the numbers of INN for central nervous system and cardiovascular medicines during the past decades. 

INN: International Nonproprietary Name.

### Essential medicines

The 22nd WHO model list^28^ contained 555 individual items, with 441 of these having a unique INN. Vaccines and other immunological agents (31) are included among the 114 medicinal substances on the list for which there was no INN. The global list contained 2060 individual items, 1590 of which had a unique INN; 1483 of these 1590 are included within our 12 selected therapeutic groups.^29^

**Fig. 5:**
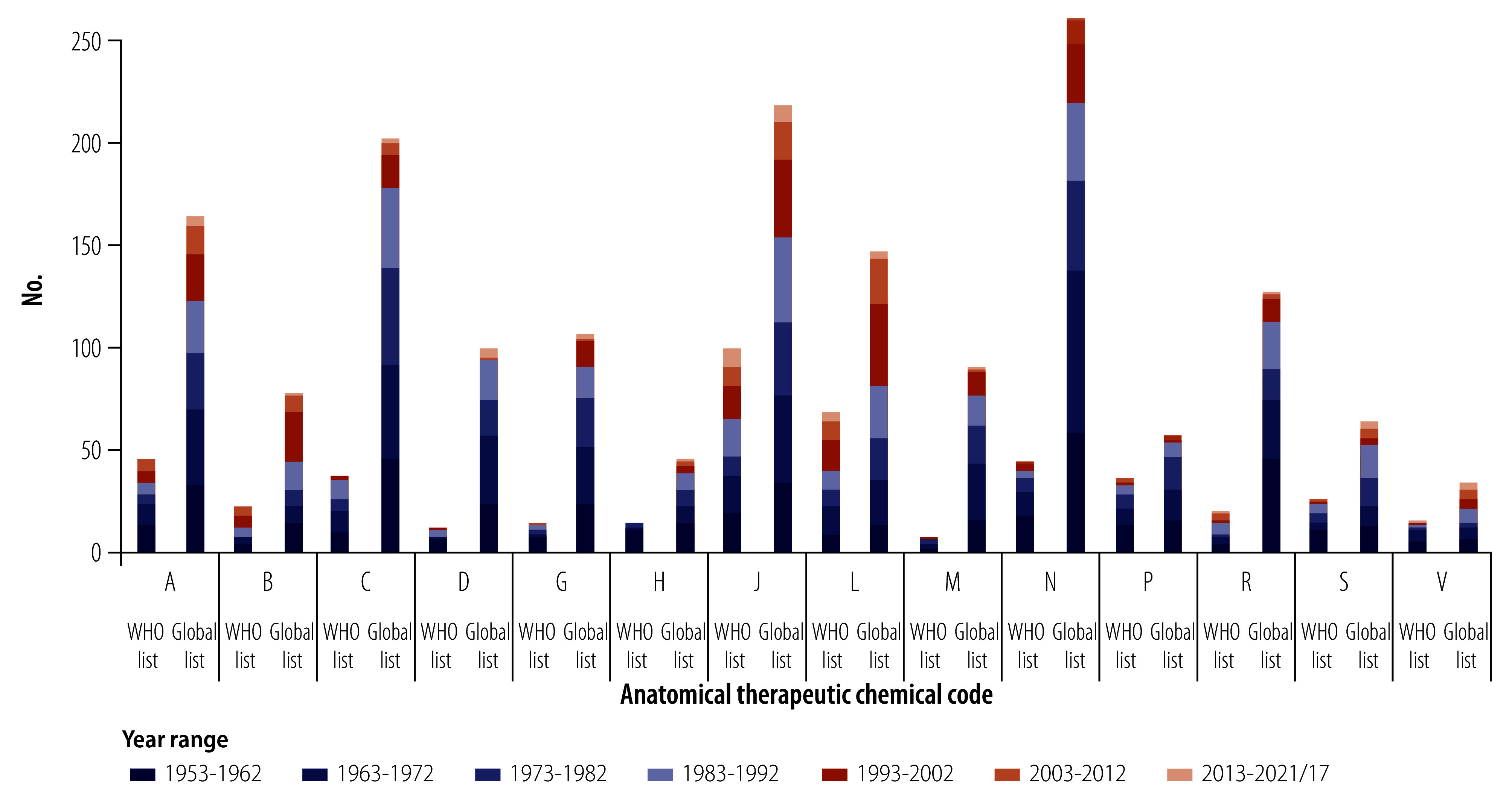
Number of INN in approved medicines categorized according to their anatomical therapeutic chemical codes, 1953–2021

INN: International Nonproprietary Name; WHO: World Health Organization.

Notes: for definition of anatomical therapeutic chemical classifications, see Norwegian Institute of Public Health.^30^ The WHO list is the 2021 *WHO Model list of essential medicines*, 1953–2021^28^ and the global list is a combined list of national essential medicines lists, 1953–2017.^29^

Fig. 5, depicting the number of INN in approved medicines that are included in the WHO model list and global list for each of the 14 anatomical therapeutic chemical classes, demonstrates a public health need for anti-infective (class J) and antiparasitic (class P) substances. These two classes comprise 30.8% (136/441) of all INN included in the 2021 WHO model list. The inclusion of anti-infective and antiparasitic substances from other classes such as alimentary tract and metabolism (class A), dermatologicals (class D), genito-urinary system and sex hormones (class G) and sensory organs (class S), increases this percentage to 34.2% (151/441). Antineoplastic and immunomodulating agents (class L) included 68 INN (15.4%), of which 45 (10.2%) are included as antineoplastic substances (class L01).

**Fig. 6:**
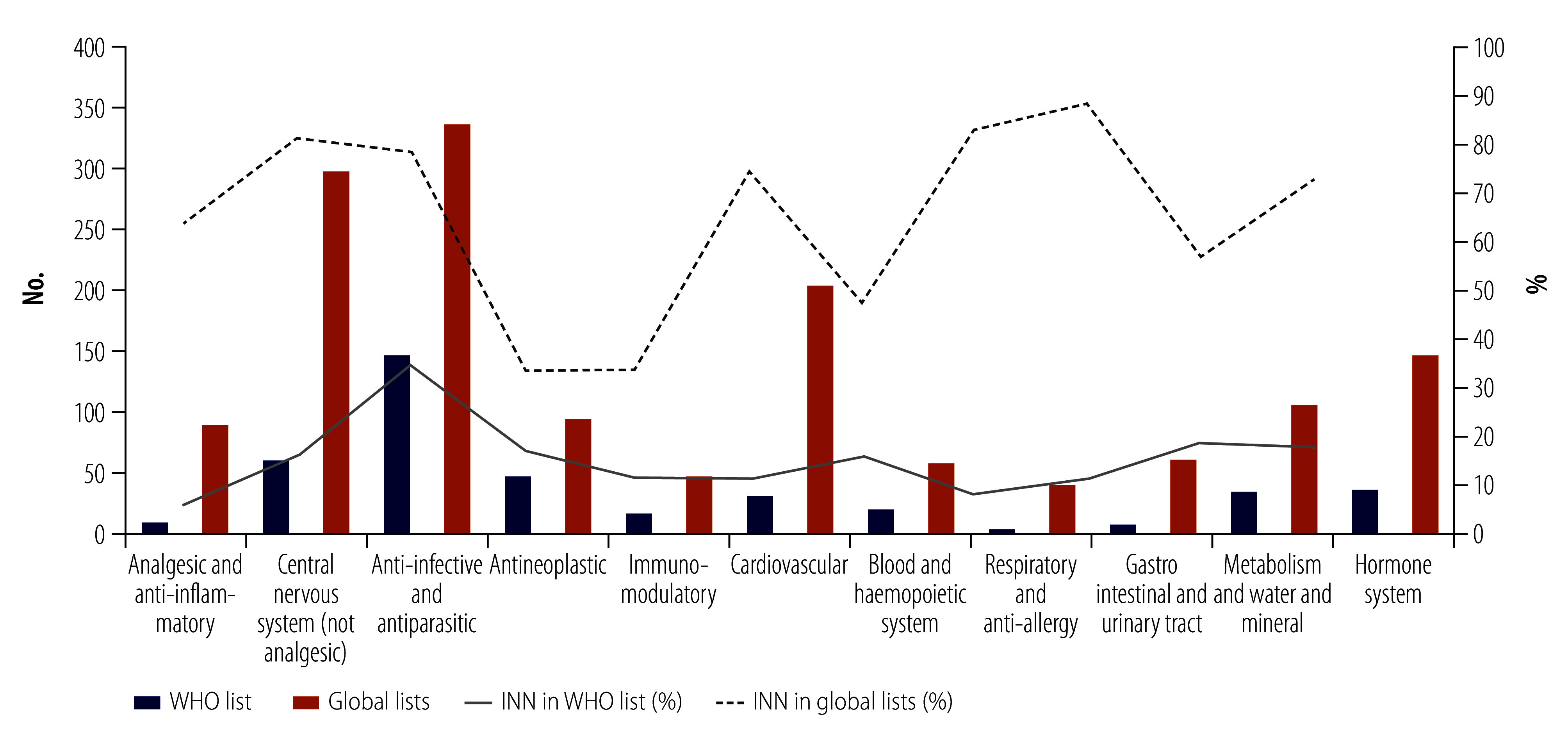
Number of INN included in the 2021 *WHO model list of essential medicines* and the global list, 1953–2021

INN: International Nonproprietary Name; WHO: World Health Organization.

Notes: the WHO list is the 2021 *WHO Model list of essential medicines*, 1953–2021^28^ and the global list is a combined list of national essential medicines lists, 1953–2017.^29^ The curves and right-hand axis provide an indication of the percentages of INN in approved medicines that are included in the respective lists. Numbers and groupings are based on INN stem classification. Only 11 out of 12 of our analysed therapeutic groups are shown because no essential medicines lists included any cell and gene therapy medicines.

In terms of public health impact, only 17.3% (441/2549) of all INN included in approved medicines (or 3.9% [441/11 453] of all INN) were included in the 22nd *WHO Model list of essential medicines*. Based on the INN stem classification, the highest proportion of 34.5% (148/429) is observed for anti-infective and antiparasitic substances, while that for antineoplastic and immunomodulatory substances are 16.7% (47/282) and 11.5% (16/139), respectively (Fig. 6). The proportion of INN in approved medicines included in global lists was 58.2% (1483/2549), with the highest proportions observed for gastrointestinal and urinary tract (88.4%; 61/69), respiratory and anti-allergy (83.3%; 40/48), and anti-infective and antiparasitic substances (78.6%; 337/429). The WHO and global lists included 9 and 27 mAbs, respectively, but it is reasonable to assume that the composition of essential medicines lists will also change in the future to reflect more technologies and pharmaceutical substances developed in recent years.

## Discussion

From the late 1960s, applications for new INN were generally published in two separate lists annually, containing between 50 and 482 INN per year. The decrease in annual INN proposed observed in the 1990s and early 2000s could be the result of changes in the research and development environment, including investment and return on investment being linked to the advent of managed care, changes in brand name protection and more rigorous regulatory requirements, market strategies, policy amendments, and global events and politics.^31^^–^^33^ The last decade of study (2013–2022) coincided with a substantial increase in new INN and thus pharmaceutical substances beginning, or progressing through, clinical trials. The benefits of technological developments, especially in the development of biological and biotechnological substances,^34^^,^^35^ as well as the increase in INN application numbers from countries such as China, contributed to this increase.^36^

The global burden of disease data demonstrate that in 2024 cardiovascular disease and cancers are the main contributors to burden of disease in higher-income countries, and that neonatal disorders, neglected tropical diseases, malaria, tuberculosis and other infectious diseases are the major contributors in lower-income countries.^37^ Based on the numbers of INN included in the selected therapeutic groups, trends in research and development are not currently aligned with unmet medical need, especially that of lower-income countries. 

The decline in new medicines for infectious and neglected tropical diseases is worrying;^2^^–^^16^ the reduction in the development of novel substances in the fields of central nervous system, cardiovascular and respiratory fields is also of concern. Substances acting on the blood and haemopoietic system peaked during the 1990s, while interest in substances acting on metabolism and water and mineral homeostasis increased in the two decades between 2003 and 2022. The first trend can be attributed to developments in the heparin, platelet aggregation and thrombin inhibitor fields. The second trend can be linked to significant development and interest in peroxisome proliferator-activated receptor and glucagon-like peptide-1 receptor agonists, dipeptidyl peptidase IV and sodium glucose co-transporter inhibitors, all of which address carbohydrate metabolism and diabetes.

The research and development trends affect the number of novel substances being registered or approved. Although the proportion of anti-infective and parasitic substances reaching the market seems to have stabilized at 13.6% (92/676) of the total INN during 2003–2022, this was the result of increased numbers of antiviral substances (66). The last antiprotozoal agent approved or registered was pyronaridine (named in 2007) and, before that, tafenoquine and artenimol (named in 1998 and 1999, respectively). No new anthelmintic or antinematode agents have reached the market since moxidectin (INN proposed in 1990), initially indicated for veterinary use and approved for human use and treatment of onchocerciasis (river blindness) in 2018.^38^

With the time to progress from phase I/II clinical trials to approval reported to be 7–8 years (although eight out of the 24 INN proposed for severe acute respiratory syndrome coronavirus 2 immunization during 2020–2022 reached market by the end of 2022),^21^^,^^35^ the bulk of pharmaceutical substances with INN proposed in the last decade of the study are currently still in the drug development pipeline. A substantial increase in antineoplastic, immunomodulatory and other biological or biotechnological medicines can therefore be expected in the next few years. 

In terms of public health impact, less than one fifth of INN were included in the 22nd *WHO Model list of essential medicines*. The largest group of medicines included is for infective diseases. Comparing lists, the WHO list contained between one tenth (analgesic and anti-inflammatory, and respiratory and anti-allergy) and one half (antineoplastic) of the approved medicines in the global list. Other disease areas where large variations are observed between the lists are the gastrointestinal and urinary tract, cardiovascular, central nervous system (not analgesic) and hormone groups.

The falling numbers of novel anti-infective and antiparasitic substances, especially antifungals, do not address neglected tropical diseases or the growing dilemma of antimicrobial resistance. The outlook for new drugs for noncommunicable diseases is equally poor, as demonstrated by the decline in research and development interest in these fields, the exploitation of well-known therapeutic targets, and the fact that any new developments are focusing on fewer indications for use in the treatment of relatively small populations.^39^ The relatively successful low-cost treatment of chronic noncommunicable diseases might also have led to a demand for medicines offering only incremental benefits.^40^

Unmet medical needs and neglected diseases have undoubtedly benefitted from new drug development technology, but novel chemical entities and additional technology remain critical. Partnerships, incentives and innovative initiatives remain of utmost importance as, with the exception of dengue and other viral neglected tropical diseases, no significant developments in this field are evident among the major pharmaceutical companies.^8^ The environment for development of medicines for neglected diseases remains dependent on product development partnerships such as the Drugs for Neglected Diseases initiative (DNDi),^41^ Medicines for Malaria, academic laboratories, and investments such as the Bio Ventures for Global Health accelerator. Regulatory incentives such as the priority review voucher and orphan products grant programmes of the United States Food and Drug Administration (FDA) have seen limited success in this field, with only 4.4% (37/850) of new registered products approved (comprising 25 products with new formulations or indications, and 8 vaccine or biological products). Only 1.2% (4/336%) of new medicines approved for neglected diseases between 2000 and 2011 contained new chemical entities.^42^ Initiatives such as Generating Antibiotic Incentives Now have also been ineffective in meeting the need for new chemical entities with antimicrobial actvity.^43^

However, although limited on the macroscale, studies indicate that 2.4 billion people have benefitted from more than 60 new health technologies introduced by product development partnerships.^44^ Two examples of successful neglected tropical disease initiatives are (i) the 2018 FDA approval of moxidectin for onchocerciasis (river blindness) via a collaboration between Medicines Development for Global Health (who received a priority review voucher), WHO Special Programme for Research and Training in Tropical Diseases, Medicines for Malaria Venture and GlaxoSmithKline;^45^ and (ii) the development of tafenoquine for *Plasmodium vivax* by GlaxoSmithKline (who received a priority review voucher) in collaboration with Medicines for Malaria Venture.^38^ Although INN for these substances date from 1989 and 1998, respectively, the collaborations and priority review vouchers contributed to getting these medicines to market. Another example is fexinidazole, the INN of which was proposed in 1977. Although initially developed as a broad-spectrum antimicrobial, fexinidazole is now approved for African trypanosomiasis and included on the *WHO Model list of essential medicines* as a result of the DNDi and Sanofi collaboration.^46^

The repurposing of existing substances has also been shown to be an effective approach to treating neglected tropical diseases. Pertinent examples include: miltefosine, an antineoplastic substance from the 1980s, now approved for the treatment of visceral leishmaniasis;^47^ amphotericin B, used for treating fungal infections since the 1960s and now also in the treatment of leishmaniasis;^48^ and eflornithine, developed as an antineoplastic and approved in 1990 for the treatment of African trypanosomiasis.^49^

A limitation of our study is that the INN stem classification is based on the mechanism or mode of action as submitted by the applicant for an INN; in some instances, the final therapeutic application, or even the proposed mode of action, could change during the development process or as a result of the substance being repurposed at a later stage. Classification by anatomical therapeutic chemical code is only available for medicines marketed in regions where this is a requirement. It would therefore be beneficial if a method of harmonizing the INN Programme and anatomic therapeutic classification could be realized.

Our study has highlighted that, despite various initiatives to address neglected diseases and multiple programmes focusing on antimicrobial resistance and stewardship, there has been no significant growth in drug discovery and development in these fields over the past few decades. This absence of important new medicines, combined with a decrease in novel therapies for high-incidence noncommunicable diseases, indicates that current and probable future global needs are not being met. We conclude that research and development strategies and investments require better alignment with global medical needs.
